# Torpor-like Hypothermia Induced by A1 Adenosine Receptor Agonist: A Novel Approach to Protect against Neuroinflammation

**DOI:** 10.3390/ijms241311036

**Published:** 2023-07-03

**Authors:** Kang Fu, Chunlei Hui, Xinyuan Wang, Tingting Ji, Xiuqing Li, Rui Sun, Chunlei Xing, Xi Fan, Yuanqing Gao, Li Su

**Affiliations:** 1 Key Laboratory of Cardiovascular and Cerebrovascular Medicine, School of Pharmacy, Nanjing Medical University, Nanjing 211166, China; 2 Institute of Translational Medicine, Shanghai University, Shanghai 200444, China

**Keywords:** hypothermia, A1 adenosine receptor, blood–brain barrier, neuroinflammation, torpor, N6-cyclohexyladenosine

## Abstract

Hypothermia is a promising clinical therapy for acute injuries, including neural damage, but it also faces practical limitations due to the complexities of the equipment and procedures required. This study investigates the use of the A1 adenosine receptor (A1AR) agonist N6-cyclohexyladenosine (CHA) as a more accessible method to induce steady, torpor-like hypothermic states. Additionally, this study investigates the protective potential of CHA against LPS-induced sepsis and neuroinflammation. Our results reveal that CHA can successfully induce a hypothermic state by activating a neuronal circuit similar to the one that induces physiological torpor. This state is characterized by maintaining a steady core body temperature below 28 °C. We further found that this torpor-like state effectively mitigates neuroinflammation and preserves the integrity of the blood–brain barrier during sepsis, thereby limiting the infiltration of inflammatory factors into the central nervous system. Instead of being a direct effect of CHA, this protective effect is attributed to inhibiting pro-inflammatory responses in macrophages and reducing oxidative stress damage in endothelial cells under systemic hypothermia. These results suggest that A1AR agonists such as CHA could potentially be potent neuroprotective agents against neuroinflammation. They also shed light on possible future directions for the application of hypothermia-based therapies in the treatment of sepsis and other neuroinflammatory conditions.

## 1. Introduction

Hypothermia has emerged as a promising clinical therapy for systemic inflammation by reducing metabolic demand, inhibiting pathogen growth, and decreasing inflammation, which collectively contributes to limiting tissue damage and promoting recovery [1,2]. During the recent COVID-19 pandemic, several case reports used therapeutic hypothermia to prevent hypermetabolism and cytokine storms [3,4]. Therapeutic hypothermia has been found particularly beneficial for neuroprotection in traumatic brain injury, cardiac arrest, and stroke cases. It has been shown to reduce neuronal damage and improve functional outcomes by suppressing the release of excitotoxic neurotransmitters, decreasing oxidative stress, and reducing apoptotic cell death [5,6]. However, systemic hypothermia therapy requires sophisticated equipment and complex procedures, limiting its applicability [7]. Thus, an urgent need is to explore alternative methods or identify safe drugs that can induce hypothermia more conveniently.

The investigation of hypothermic therapy has drawn inspiration from the phenomenon of hibernation. Hibernation is a unique phenomenon characterized by reducing an animal’s core body temperature and metabolic rate to conserve energy [8,9,10]. Non-hibernating mammals, such as mice, can also exhibit hibernation-like states called “torpor” for a few minutes to hours under certain conditions, such as prolonged fasting in cold ambient temperatures [11,12]. Neural circuits controlling these hibernation-like states and the compounds capable of inducing torpor have become focal points in the field. The interest stems from the potential therapeutic benefits of inducing natural torpor in non-hibernating mammals and humans, as it may provide similar therapeutic effects as hypothermia with minimal side effects. Activation of specific neurons by chemical, genetic, or optogenetic approaches and some pharmacological methods have been reported to induce torpor-like hypothermic states in non-hibernating mammals. These findings may have significant implications for critical care treatment [13,14,15].

The A1 adenosine receptor (A1AR) agonist N6-cyclohexyladenosine (CHA) has been demonstrated to induce a steady torpor-like state in non-hibernating rats, exhibiting hypothermia, a lower heart rate, and reduced arterial pressure [16,17]. In this study, we explored the potential protective effects of the A1AR agonist CHA as a torpor inducer against LPS-induced neuroinflammation. Consistent with the previous literature, we found that CHA rapidly reduced core body temperature and induced a torpor state while decreasing LPS-induced neuroinflammation. Furthermore, we investigated the neural circuits involved in CHA-induced torpor and the possible mechanisms underlying its neuroprotective effects. The CHA-activated neuronal circuit is comparable to natural torpor. The protective effect of CHA was achieved by reducing the permeability of the BBB and thereby preventing the entry of LPS-induced inflammatory factors into the central nervous system. Direct activation of A1AR on immune or endothelial cells has little impact on LPS-induced inflammatory responses and blood–brain barrier leakage. The protective effect is mainly mediated by neuronal-based systemic hypothermia. Thus, our data demonstrate that A1AR agonists exhibit significant neuroprotective effects against LPS-induced neuroinflammation by inducing torpor. This finding holds substantial clinical implications for the future application of hypothermia-based therapies for sepsis and other infection-related neuroinflammatory diseases.

## 2. Results

### 2.1. The (A1AR) Agonist N6-cyclohexyladenosine (CHA) Induces a Stable Torpor State upon LPS Infection in Mice

**Figure 1:**
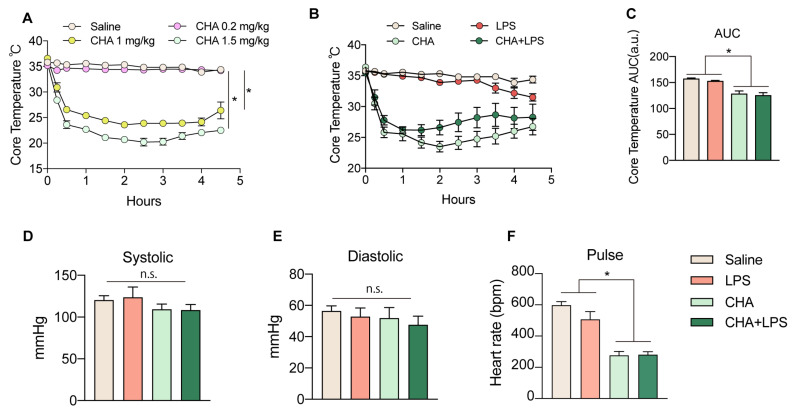
CHA induces a stable torpor state upon LPS infection. (**A**) The core temperature of mice received i.p. injection of saline and three dosages of CHA (0.2 mg/kg, 1 mg/kg, and 1.5 mg/kg). The mice received an i.p. injection of 1.5 mg/kg of CHA, together with or without 4 mg/kg LPS. The core temperature was recorded in (**B**), and the area under the curve of (**B**) was plotted in (**C**). The systolic pressure (**D**), diastolic pressure (**E**), and heart rate (**F**) were also recorded in all four groups. CHA: N6-cyclohexyladenosine. n.s. = not significant. *p* < 0.05 for *.

First, we investigated the effects of different doses of CHA on the induction of a torpor state after administering it through systemic intraperitoneal (i.p.) injection. We found 1 mg/kg and 1.5 mg/kg of CHA efficiently and consistently induced a torpor-like state in non-hibernating mice, which persisted for a minimum of 4–6 h (Figure 1A). After administration, there was a rapid decline in core body temperature within 15 min that remained between 22–25 °C. Since some mice treated with a dose of 1 mg/kg exhibited a temperature rebound after 4 h, we proceeded with the 1.5 mg/kg dose for subsequent experiments. To explore the potential protective effects of CHA against LPS-induced neuroinflammation, we administered LPS to mice to induce acute sepsis, together with CHA, right before LPS. We closely monitored changes in body temperature within six hours of administration. Firstly, even during the course of LPS-induced sepsis, CHA consistently induced a torpor-like state (Figure 1B,C). Furthermore, throughout this process, there were no significant alterations in systolic and diastolic blood pressure (Figure 1D,E), while the heart rate of CHA-treated mice was significantly decreased. (Figure 1F).

### 2.2. CHA Protects against LPS-Induced Neuroinflammation and Microgliosis

**Figure 2:**
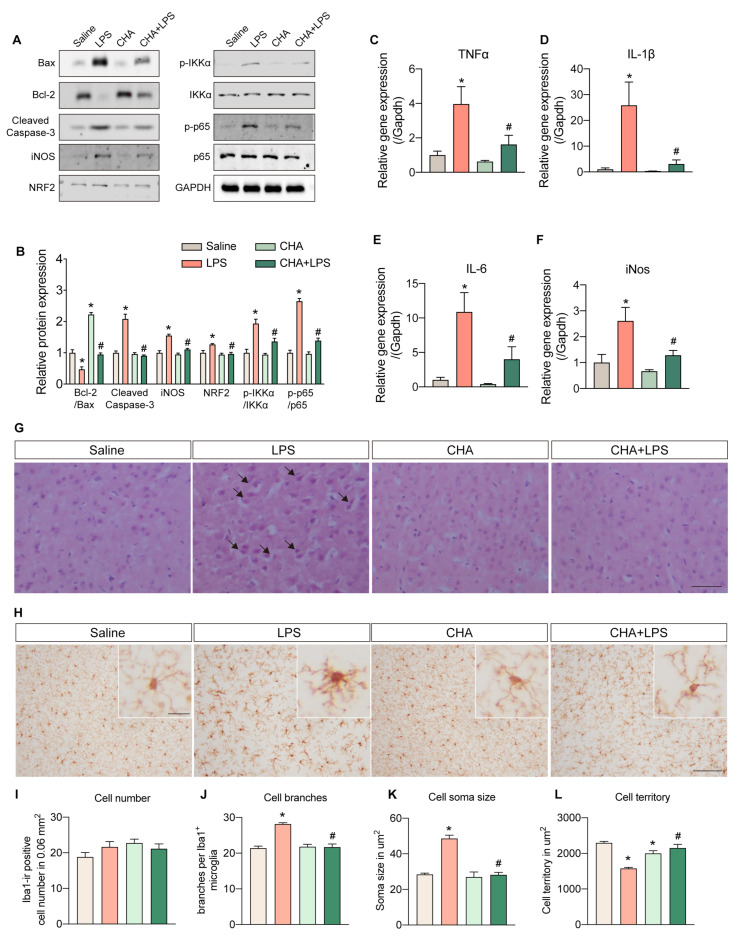
CHA protects against LPS-induced neuroinflammation and microgliosis. (**A**) Protein levels of Bax, Bcl-2, cleaved caspase-3, iNOS, NRF2, p-IKKα, IKKα, p-p65, and p65 among four groups. (**B**) quantification of the protein band intensity of (**A**). (**C**–**F**) mRNA levels of pro-inflammatory cytokines (TNF-α, IL-1β, IL-6, and iNos) in the brain among four groups. (**G**) Representative histopathological images from H&E staining of the cortex among four groups. (**H**) The morphology of microglia is evidenced by anti-Iba1 staining. The microgliosis was quantified by cell number (**I**), branches per cell (**J**), soma size (**K**), and cell territory (**L**). Black arrow in (**G**): swelling and vacuolated neuron. *p* < 0.05 for * vs. the saline group. *p* < 0.05 for # vs. LPS group. Scale bar = 100 µm in (**G**) and (**H**), 10 µm in amplified (**H**).

We next investigated the potential protective role of CHA in LPS-induced neuroinflammation. Key apoptosis and inflammatory signaling markers were examined at the protein and mRNA levels in brain tissue. The Bcl-2/Bax ratio was significantly decreased in the LPS group, indicating activation of apoptotic signaling. The cleaved caspase-3 level was markedly increased, further supporting apoptotic activation. Remarkably, CHA intervention significantly attenuated these alterations, suggesting its ability to prevent the apoptotic process (Figure 2A,B). Inflammatory markers such as iNOS, NRF2, and phosphorylation levels of IKKα and p65 were significantly upregulated in response to LPS stimulation. The CHA intervention also effectively mitigated the inflammatory responses. Moreover, the mRNA levels of TNF-α, IL-1β, IL-6, and iNOS in the brain exhibited substantial upregulation in the LPS group and were significantly reduced upon CHA intervention (Figure 2C–F).

Histological examination using H&E staining revealed the presence of swelling and vacuolated neurons in the LPS-treated brain cortex, which was not observed in the other experimental groups (Figure 2G). As microglia are resident immune cells in the brain, we also analyzed their morphological changes. LPS induction led to evident microgliosis, characterized by increased cell branching, enlarged soma size, and reduced cell territory. Remarkably, CHA administration significantly rescued these inflammatory responses (Figure 2H–L). Collectively, these data demonstrate that CHA exerts a pronounced protective effect against LPS-induced neuroinflammation.

### 2.3. CHA Prevents the LPS-Induced Blood–Brain Barrier Disruption

**Figure 3:**
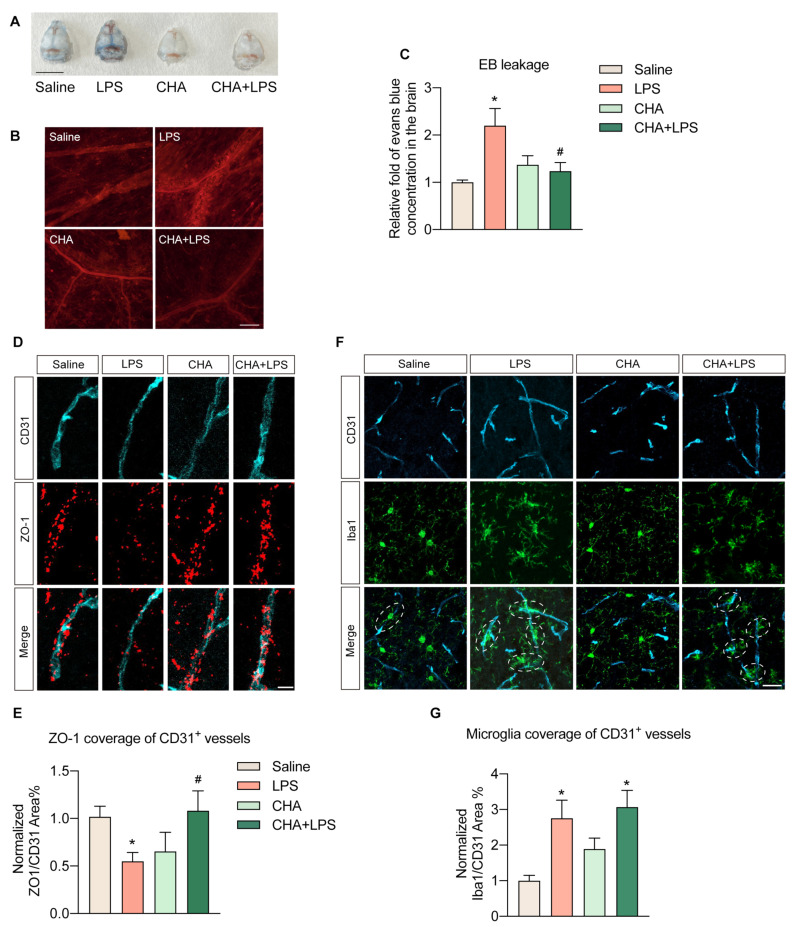
CHA reduces the LPS-induced blood–brain barrier leakage. (**A**) The photo of skulls from four groups of animals after Evans’ blue injection. (**B**) The immunofluorescence of Evans blue in the meninges of the skull. (**C**) Relative concentration of Evans blue dye extravasation into the brain of four groups. (**D**) Co-staining of anti-CD31 and anti-ZO-1. (**E**) Quantification of ZO-1 coverage of CD31^+^ vessels in (**D**). (**F**) Co-staining of anti-CD31 and anti-Iba1. (**G**) Quantification of microglia coverage in CD31^+^ vessels in (**F**). *p* < 0.05 for * vs. the saline group, *p* < 0.05 for # vs. the LPS group. Scale bar = 1 mm in (**A**), 100 µm in (**B**), 25 µm in (**D**), and 10 µm in (**F**).

Neuroinflammation and subsequent injury induced by LPS are primarily due to the compromise of the blood–brain barrier (BBB), which facilitates the infiltration of peripheral inflammatory factors and immune cells. Therefore, we further investigated the impact of CHA on the BBB. To assess the integrity of the BBB, we first injected Evans blue dye to evaluate the BBB’s permeability. A clear photograph of the brain skull displayed a notable extravasation of Evans blue in the LPS group, which was virtually non-existent in the other three groups (Figure 3A). Under a microscope, the meningeal vessels in the LPS group manifested red fluorescent signals, signifying Evans’ blue leakage beyond the vessel walls. In contrast, this phenomenon was missing in the other three groups (Figure 3B). Further quantitative analysis of Evans blue signals extracted from brain tissue by formamide verified that LPS indeed instigated substantial extravasation of Evans blue into the brain, effectively hindered by CHA intervention (Figure 3C).

We further characterized this change by co-staining the BBB marker, the tight junction protein ZO-1, with the blood vessel marker CD31. The area of brain vasculature covered by ZO-1 protein was notably diminished in the LPS group, whereas CHA treatment effectively mitigated the loss of ZO-1 (Figure 3D,E). Additionally, we validated this occurrence through the reaction of perivascular microglia. In the LPS group, there was a significant increase in the number of activated microglia interacting with brain vessels. Following CHA treatment, although the number of perivascular microglia also rose, their activity was significantly attenuated compared to the LPS group. This indicates decreased infiltration of pro-inflammatory cytokines (Figure 3F,G). Collectively, these findings propose that CHA’s anti-neuroinflammation effect might be achieved by preserving BBB integrity.

### 2.4. CHA Induces Torpor through POA-PVN-DMH-AP Neural Circuitry

**Figure 4:**
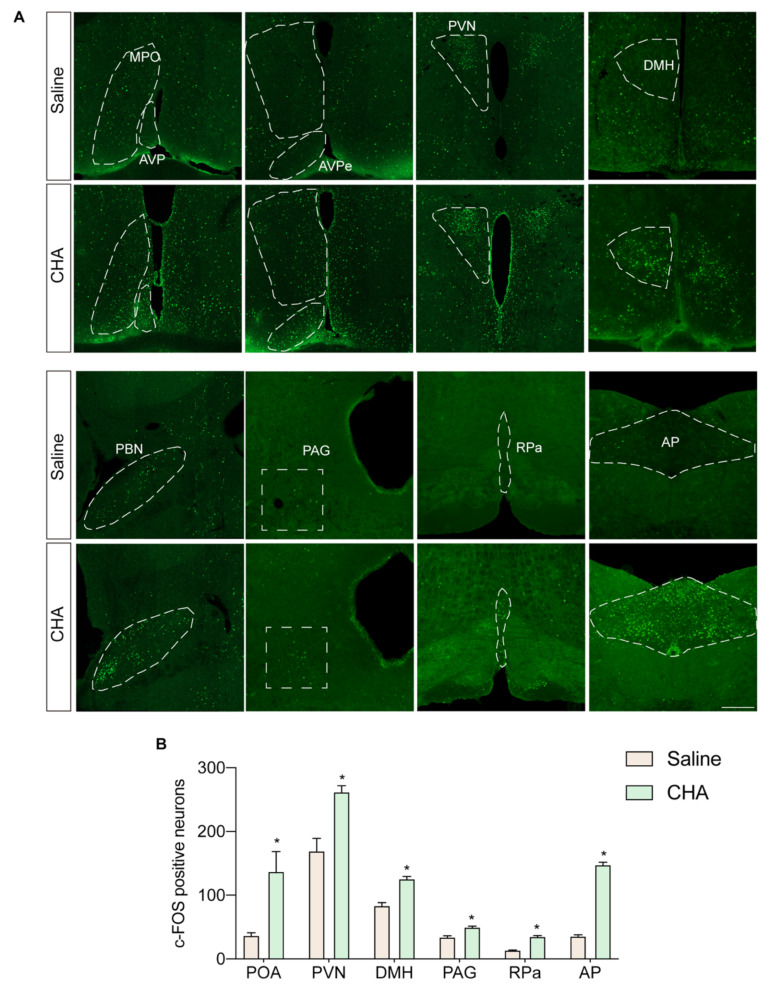
Whole brain tracing of neural circuit activated by CHA. (**A**) c-Fos staining of POA, PVN, DMH, PBN, PAG, RPa, and AP nucleus. (**B**) Quantification of c-Fos-positive neurons in (**A**). POA: preoptic area; MPO: medial preoptic area; AVP: anterior ventral preoptic area; PVN: paraventricular nucleus; DMH: dorsal medial hypothalamus; PBN: parabrachial nucleus; PAG: periaqueductal gray; RPa: raphe pallidus; AP: area postrema. *p* < 0.05 for *. Scale bar = 200 µm.

To investigate the mechanisms underlying the neuroprotective effects of CHA, we first explored the neural circuits activated during CHA-induced torpor. By tracing the c-fos signal across different anatomical locations involved in temperature and metabolism regulation, we observed strong activation of the preoptic area (POA), PVN (paraventricular nucleus), DMH (dorsal medial hypothalamus), PBN (parabrachial nucleus), and AP regions (area postrema) in response to CHA treatment (Figure 4A,B).

We also employed a prolonged fasting approach to induce spontaneous torpor in mice to compare these findings with physiologically spontaneous situations under non-pharmacological induction. After 36 h of fasting and exposure to a cool environment at 16 °C, some mice entered a transient torpor state characterized by a drop in core body temperature below 32 °C and a state of numbness and inactivity. Similarly, we traced the activated neural circuits using c-Fos staining. We found that most activated nuclei were similar to those activated by CHA, including the temperature-regulating nuclei in the POA, PVN, and DMH (Figure S1A,B). The POA region is considered a central hub for temperature regulation, and the PVN and DMH receive neuronal projections from the POA. Midbrain nuclei such as PAG, RPa, and PBN receive projections from the PVN. These nuclei further regulate the activity of the sympathetic and parasympathetic nervous systems to adjust cardiovascular function and vascular constriction in response to changes in body temperature and metabolic rate. Thus, the neural circuitry activated by CHA is highly similar to that initiated by physiologically spontaneous torpor. The main difference lies in the strong activation of the arcuate nucleus (ARC) induced by fasting, which was not observed with CHA. At the same time, CHA strongly activated the AP region, which was absent in the fasting-induced torpor scenario (Figure S1A–C).

### 2.5. CHA-Induced Hypothermia Mitigates Inflammation and Oxidative Stress in Endothelial Cells

**Figure 5:**
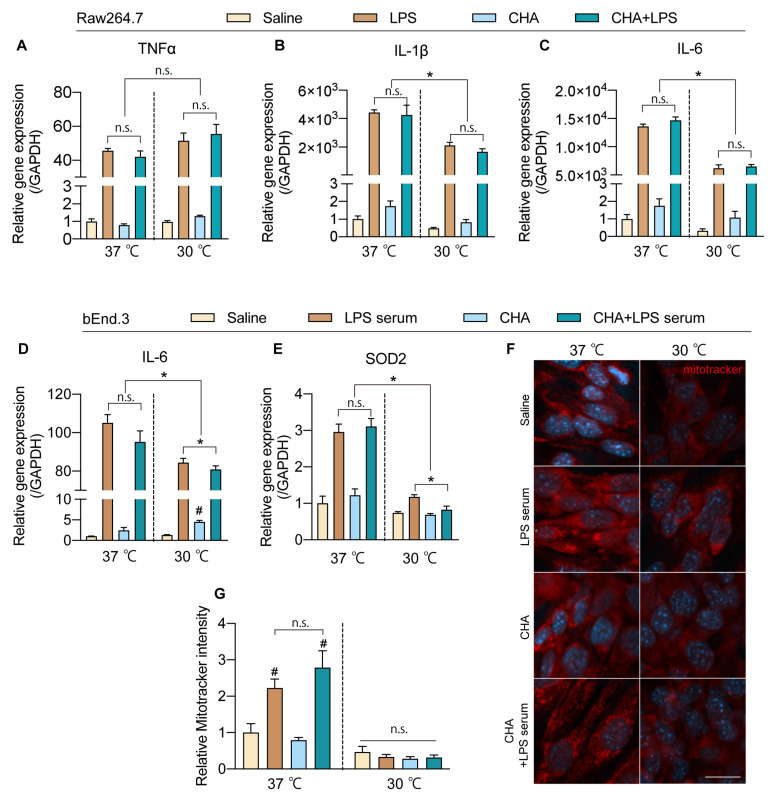
CHA-induced torpor inhibits inflammatory responses of macrophages and oxidative stress in brain endothelial cells. (**A**–**C**) mRNA levels of pro-inflammatory cytokines (TNF-α, IL-1β, and IL-6) in macrophages at 37 °C and 30 °C after LPS stimulus with or without CHA. (**D**,**E**) mRNA levels of IL-6 and SOD2 in bEnd.3 cells at 37 °C and 30 °C after LPS stimulus with or without CHA. (**F**) Mitochondria activity is evidenced by the mitotracker probe under the same settings of (**D**,**E**). (**G**) Quantification of mitotracker intensity in (**F**). n.s. = not significant. Dotted lines are just to separate two groups for easier understanding. *p* < 0.05 for *. *p* < 0.05 for # vs. the saline group. Scale bar = 10 µm in (**F**).

According to the database, although the predominant expression of A1AR was limited to the brain, sporadic reports have suggested that A1AR agonists can directly act on macrophages to inhibit pro-inflammatory responses. To further elucidate whether the protective effects of CHA occur directly through actions on immune cells and endothelial cells or indirectly through CHA’s induction of hypothermia, we simulated the inflammatory stimulation caused by LPS in microglia, macrophages, and brain vascular endothelial cells. Then, we observed the inflammatory response and oxidative stress levels of these cells after intervention with hypothermia or CHA. Treatment with CHA did not alter the inflammatory response of microglia to LPS (Figure S2A) or affect the inflammatory response of RAW264.7 macrophages to LPS (Figure 5A–C). In contrast, low-temperature treatment significantly inhibited the mRNA levels of IL-1β and IL-6 in macrophages stimulated by LPS, with no apparent impact on TNF-α (Figure 5A–C). In bEND.3 cells, following stimulation with 4% inflammatory serum from septic mice in the culture medium, the levels of TNF-α and IL-1β did not show significant changes (Figure S2B). In contrast, the level of IL-6 increased significantly. This response was not affected by CHA but could be attenuated by hypothermia (Figure 5D). On the other hand, the expression of Superoxide Dismutase 2 (SOD2), a key enzyme involved in mitochondrial oxidative stress, was significantly upregulated in response to inflammatory serum stimulation, and CHA did not directly affect this process. Similarly, hypothermia significantly reduced the elevation of SOD2 induced by inflammatory serum stimulation (Figure 5E). Mitotracker staining, indicative of mitochondrial activity, also showed a similar pattern. CHA did not affect the increased mitochondrial activity after inflammatory serum stimulation, but hypothermia significantly suppressed mitochondrial activity levels (Figure 5F,G). Collectively, these data suggest that the protective effects of CHA on neuroinflammation and endothelial cells are not mediated through direct actions on A1AR receptors in these cells. Instead, the effects are likely attributed to CHA’s activation of the thermoregulatory neurocircuitry, inducing hypothermia. Subsequently, hypothermia plays a crucial role in reducing inflammatory responses and oxidative stress damage in endothelial cells.

## 3. Discussion

In this study, we discovered that the A1AR agonist CHA could rapidly and consistently induce a torpor-like state in non-hibernating mice. Even under conditions of systemic inflammation induced by LPS, CHA maintains stable hypothermia, effectively protecting the integrity of the blood–brain barrier and reducing neuroinflammatory damage caused by LPS. This protective effect is primarily achieved through reducing inflammatory responses and endothelial oxidative stress under hypothermia rather than through the direct actions of CHA. Therefore, CHA can potentially be an effective therapeutic drug for treating infections and critical illnesses.

Our data suggest that the protective effect of CHA on blood–brain barrier integrity is likely the main mechanism underlying its protection against neuroinflammation. Previous studies have indicated that hyperthermia increases BBB permeability, while therapeutic hypothermia can have a protective effect [18,19,20,21,22]. Consistent with the literature, our data also confirm that CHA-induced hypothermia significantly prevents the loss of zo-1, preserving blood–brain barrier integrity. It is worth noting that systemic inflammation induced by LPS is often accompanied by fever, which may exacerbate the damage to the blood–brain barrier caused by inflammation [21]. Fever is a process initiated by the hypothalamic region (POA) in response to infection, activating the immune system and serving as an innate defense mechanism of the body [23]. However, severe infections, such as sepsis, can have fatal consequences. By acting on the POA region, CHA suppresses this inflammatory positive feedback loop, protecting against severe infections.

We found that the molecular mechanism underlying the protective effect of CHA is mostly due to its indirect effect on hypothermia rather than its direct effect on A1AR receptors on endothelial and immune cells. The Human Protein Atlas database and literature indicate that A1AR is mainly expressed in inhibitory neurons and oligodendrocyte precursor cells, with minimal expression in peripheral tissues such as cardiomyocytes and hepatocytes [24,25,26,27]. Both endothelial cells and macrophages express temperature-sensitive receptors that can sense temperature changes [28,29,30]. Our data demonstrated that hypothermia could reduce the inflammatory response of macrophages and the oxidative stress response of endothelial cells, which could contribute to protecting blood–brain barrier integrity. Moreover, we also observed that the expression of TNF-α in macrophages is not affected by temperature. This indicates that cells maintain normal cellular functions under low temperatures and do not undergo apoptosis or overall inhibition of cell activity. This also indicates that immune responses and temperature sensing in macrophages may involve more complex and precise regulatory mechanisms, which require further investigation. Adenosine is recommended as a first-line antiarrhythmic drug for supraventricular tachycardia in the cardiovascular system in international guidelines [31,32]. It lowers the sinus node’s automaticity and inhibits sinus atrial conduction. This plays an important protective role in myocardial ischemia-reperfusion injury. Our study also found that CHA significantly reduces heart rate without affecting blood pressure, which may result from the combined effects of central and peripheral cardiac A1AR receptors. However, this also suggests that, in the presence of cardiovascular diseases, further attention should be paid to the potential cardiovascular side effects of CHA-induced hypothermia.

The mechanism by which CHA induces hypothermia has not been fully elucidated. Previous studies have shown that central administration of CHA to rats activates the NTS nucleus, inhibiting thermogenesis in brown adipose tissue [16]. In this study, we first revealed the whole brain neurocircuitry underlying CHA-induced torpor. We compared it with the neurocircuitry triggered by spontaneous torpor in mice in response to hunger and cool environments. We found that the circuitry activated by CHA is very similar to the circuitry involved in spontaneous torpor. Both pathways originate from the thermoregulatory core nucleus POA and project to the PVN and DMH. The PVN and DMH regulate downstream sympathetic and parasympathetic output, modulating body temperature and thermogenesis to induce torpor in mice. Furthermore, compared to torpor induced by prolonged fasting, the neurocircuitry activated by CHA does not involve the ARC. This avoids the sensation of hunger and the associated side effects of hypoglycemia and metabolic disturbances. Meanwhile, the literature reports that CHA-induced hypothermia partially operates through the A3AR signaling pathway. Additionally, the expression of A1AR is not limited to central neurons. It is also richly expressed in peripheral neurons and the heart and may contribute to torpor induction. Clarifying the targets and selectivity of CHA would assist in further diminishing unnecessary side effects in future studies.

The literature has reported that all four receptors of adenosine are involved in adenosine-induced hypothermic effects. Moreover, there is some complementary effect between these receptors [33]. Due to different receptor distributions, their mechanisms are not identical. Hypothermia via A3AR is caused by agonism of peripheral mast cell A3AR, causing histamine release, which produces hypothermia via central histamine H1 receptors [17]. Adenosine A2A agonists cause hypothermia via peripheral receptors, causing vasodilation and heat loss. Adenosine A2B agonists cause hypothermia via central action, activating POA and PVN hypothalamic neurons [34]. However, the expression of the A2B receptor is primarily enriched in glial cells rather than neurons, and there are some contradictory reports on its role in neuroinflammation [35]. Its mechanism may not be the same as the activation of A1AR. We do not rule out that other hypothermic inducers and adenosine receptor agonists may also have a similar positive effect on the blood–brain barrier. This requires further experimental evaluation in the future. Meanwhile, the other three adenosine receptors were also involved in modulating neuroinflammation [35,36,37,38]. The combined application of CHA and A2a receptor agonists could potentially offer superior neuroinflammatory protection.

By far, activating specific neurons in the POA, such as neurons positive for QRFP receptors, TRPM2, and adcyap1, can precisely induce hibernation-like torpor in mice [13,14,39]. The neurocircuitry reported in this literature is also very similar to the circuitry activated by CHA in our experiments. A1 receptors are generally coupled to Gi, reducing cAMP and conducting inhibitory signaling. The effect of A1 receptor activation on inducing hypothermia mainly relies on neuronal signaling. It is worth investigating whether there is an overlap between these neurons and neurons expressing A1AR. These findings all indicate that CHA-induced torpor closely resembles a physiological state, representing a spontaneous modulation of the temperature set point and whole-body metabolism. This is different from clinical therapeutic hypothermia, which was induced by a passive approach [40]. This voluntary approach, such as CHA induction, may allow the organism to adapt better to hypothermia, avoiding potential cold-induced damage or other side effects. Compared to methods such as optogenetics and ultrasound stimulation used to induce hypothermia in non-hibernating animals, CHA exhibits significant advantages in terms of ease of administration and rapid efficacy [41]. With further formulation improvements, such as intranasal spray delivery, it may be possible to further minimize peripheral off-target effects and achieve more precise and rapid therapeutic effects. This makes it valuable in critical care and neuroinflammation therapy.

This study does have certain limitations. We primarily examined the central neural circuits triggered by CHA. Nonetheless, A1AR receptors are also manifested in the heart and peripheral neurons. Therefore, a question emerges: Is the peripheral signaling of these A1ARs essential for inducing torpor? Moreover, further research is needed to determine whether blocking these peripheral signals could potentially benefit the cardiac system. Additionally, the mechanisms by which hypothermia exerts a protective effect on the blood–brain barrier are not fully understood. Future research is required to confirm whether different torpor inducers have comparable benefits for the blood–brain barrier and neuroinflammation. Moreover, our study primarily focused on the preventive and protective effects of neuroinflammation. However, in practical scenarios, interventions often occur post-inflammation. Hence, it is worth exploring whether torpor remains effective once inflammation and disease stress models have set in and how it performs when used with other anti-inflammatory drugs.

## 4. Materials and Methods

### 4.1. Animals

Eight to ten-week-old C57BL/6 male mice were used in this study. The mice have ad libitum access to a standard diet and sterilized water based on a 12 h dark–light cycle. Animals were euthanized by isoflurane overdose (adult mouse), confirmed by the removal of the brain, in accordance with the protocol approved in the Animals (Scientific Procedures) Acts 1986. All experimental procedures conformed to guidelines and protocols approved by the Ethics Committee of Shanghai University (Approval No. ECSHU2021-169) and followed the guidelines for the National Institute of Health Guide for the Care and Use of Laboratory Animals.

### 4.2. Torpor Induction and Experimental Groups

To induce torpor status by fasting, mice were subjected to a 36-h fasting period starting at 7 p.m. on day 1 and lasting until 7 a.m. on day 3. Following the fasting period, the mice were kept in a cool environment with a room temperature of 16 °C for two hours. Torpor was defined as a body core temperature below 32 °C. To induce a stable torpor status by pharmacological approach, mice received intraperitoneally (i.p.) injections of N6-cyclohexyladenosine (CHA) (HY-18939, MCE, Shanghai, China) at dosages with saline as solvent: 0.2 mg/kg, 1 mg/kg, and 1.5 mg/kg. The room temperature is 22 °C. The rectal temperature was monitored continuously for 4–5 h. 1.5 mg/kg was selected in the later experiment. To test the protective effect of CHA against LPS-induced neuroinflammation, animals were divided into four groups: saline, CHA, LPS (L2630, Sigma-Aldrich, St. Louis, MO, USA), and LPS, together with CHA. A total of 4 mg/kg LPS were injected intraperitoneally (i.p.) to induce sepsis [42]. A total of 1.5 mg/kg CHA were i.p. injected 30 min prior to LPS to induce torpor. Organs were harvested 6 h after LPS injection. Rectal temperature was measured by a digital animal thermometer and recorded every 30 min (n = 8 for each group).

### 4.3. Core Temperature Measurement

Rectal temperature was used to represent the body’s core temperature [43]. Measurements were obtained using a digital animal thermometer (TH-212, Hendajin Co., Guangzhou, China). The thermometer probe was inserted 1.5 cm into the rectum and held in place for 20 s until a stable temperature reading was obtained.

### 4.4. Assessment of Blood–Brain Barrier Permeability

Blood–brain barrier (BBB) permeability was assessed by leakage of Evans blue, as previously described [44]. Briefly, 2% Evans blue (206334, Sigma-Aldrich, St. Louis, MO, USA) was i.p. injected at 4 mL/kg into each mouse two hours before sacrifice. A piece of cortex tissue was weighted and then incubated in 300 µL formamide at 55 °C for 24 h. On the following day, the samples were centrifuged at 4 °C for 30 min at 12,000 g. The supernatant was collected for analysis. The Evans blue extravasation was evaluated by measuring the optical density (O.D.) at 620 nm with a spectrophotometer.

### 4.5. Cell Culture

The mouse macrophage Raw264.7 cells were cultured in RPMI 1640 medium (C11875500BT, Gibco, Shanghai, China) supplemented with 100 U/mL penicillin and streptomycin (15140122, Gibco, Shanghai, China), as well as 10% fetal bovine serum (FBS) (10099141, Gibco, Shanghai, China). When the cells reached 90% confluency, they were treated with either 10 µM CHA or saline in the presence or absence of 100 ng/mL LPS for six hours. Half of the cells were maintained in a 30 °C incubator. The mouse microglia BV2 cell line was cultured in DMEM medium (C11995500BT, Gibco, Shanghai, China) supplemented with 10% FBS, penicillin, and streptomycin. Similar to Raw264.7 cells, BV2 cells were prepared for the experiment and treated in the same manner as Raw264.7 cells. The mouse brain endothelial bEnd.3 cells were also cultured using the same medium as Raw264.7 cells. These cells were maintained in a humidified 5% CO_2_ incubator at 37 °C. When the cells reached 90% confluency, they were treated with either 10 µM CHA or saline, along with a 4% sterilized serum obtained from mice injected with LPS (referred to as LPS-serum). The cells were then incubated at 37 °C and 30 °C for a period of six hours, respectively. Mitochondria activity was assessed by the MitoTracker Red CMXRos probe (Yeasen, 40741ES50).

### 4.6. Real-Time Quantitative PCR

Total RNA was isolated from tissues or cells by Trizol (Vazyme, Nanjing, China) and reverse-transcribed into cDNA using the HifaiII 1st Strand cDNA Synthesis Kit (Yeasen, Shanghai, China). Real-time RT-PCR was executed with SYBR Green Master Mix (Yeasen, Shanghai, China). The expression of target genes was normalized to glyceraldehyde-3-phosphate dehydrogenase (Gapdh) and quantified with the 2^−ΔΔCT^ method. The primers used are as follows; Sod2: forward, 5′- GTGGAGAACCCAAAGGAGAGTT-3′, reverse, 5′-CAGGCAGCAATCTGTAAGCG-3′; iNos: forward, 5′-TCTAGTGAAGCAAAGCCCAA CA-3′, reverse, 5′-TGATGGACCCCAAGCAAGAC-3′; IL-1β: forward, 5′-TGCCACCTTTTGACAGTGATG-3′, reverse, 5′- AAGGTCCACGGGAAAGACAC-3′; IL-6: forward, 5′-CCAGTTGCCTTCTTGGGACT-3′, reverse, 5′-GGTCTGTTGGGAGTGGTATCC-3′; TNF-α: forward, 5′-CCCAAAGGGATGAGAAGTTCC-3′, reverse, 5′-GCTACAGGCTTGT CACTCGAA-3′.

### 4.7. Western Blotting

Tissues and cells were homogenized using RIPA lysis buffer supplemented with a protease inhibitor cocktail. Following centrifugation, the protein concentration in the resulting supernatant was determined by the BCA Protein Concentration Kit. The denatured protein samples were then separated on a 10% SDS-PAGE gel and transferred onto PVDF membranes. The PVDF membranes were blocked in TBS-T (containing 0.1% Tween 20 and 5% BSA) to prevent nonspecific binding for 90 min. Subsequently, the PVDF membranes were cut according to the corresponding molecular weights and incubated overnight at 4 °C with specific primary antibodies, including GAPDH (2118S, Cell Signaling Technology, Danvers, MA, USA), Bax (41162S, Cell Signaling Technology, Danvers, MA, USA), Bcl-2 (3498S, Cell Signaling Technology, Danvers, MA, USA), cleaved caspase-3 (9664S, Cell Signaling Technology, Danvers, MA, USA), iNOS (AF0199, Affinity Bioscience, Cincinnati, OH, USA), NRF2 (AF0639, Affinity Bioscience, Cincinnati, OH, USA), IKKα (61294S, Cell Signaling Technology, Danvers, MA, USA), p-IKKα (2697S, Cell Signaling Technology, Danvers, USA), p65 (8242S, Cell Signaling Technology, Danvers, MA, USA), and p-p65 (3033S, Cell Signaling Technology, Danvers, MA, USA). The membranes were incubated with the appropriate secondary antibodies for two hours at room temperature the following day. The protein bands were visualized using an ultrasensitive ECL and captured by a Bio-Rad automated gel imaging system [45].

### 4.8. Immunohistochemistry and Immunofluorescence

Immunohistochemistry and immunofluorescence were conducted following our previously described methods [46]. Briefly, after euthanasia, mice were perfused transcardially with cold saline. The brain was post-fixed in 4% paraformaldehyde (PFA) for 48 h, dehydrated in a 30% sucrose solution, embedded with OCT (Sakura, Torrance, CA, USA), and cut into 30 µm cryosections. Brain sections were first incubated with primary antibodies overnight at 4 °C. Subsequently, they incubated with a biotin-conjugated secondary antibody followed by an avidin–biotin–horseradish peroxidase complex. For diaminobenzidine (DAB) staining, signals were visualized by 1% diaminobenzidine with 0.01% hydrogen peroxide. For immunofluorescent staining, sections were incubated with Alexa dye-conjugated secondary antibodies. The following primary antibodies were used in this study: anti-cFos (2250T, Cell Signaling Technology, Danvers, USA); anti-Iba1 (019-19741, Wako, Osaka, Japan); anti-CD31 (AF3628, R&D system, Minneapolis, USA); and anti-ZO-1 (SAF012, AiFang Biological, Wuhan, China). Images were captured using a fluorescence microscope (Olympus, Tokyo, Japan) or an LSM800 laser confocal microscope (Zeiss, Oberkochen, Germany).

### 4.9. Image Analysis

For quantification of ZO-1, CD31, and Iba1, immunoreactive (ir) intensity or cell number was analyzed by ImageJ software (2.0.0-rc-43/1.50e version). The immunoreactivity (ir) is represented by the area covered by ir-positive signals above the fixed threshold. The c-Fos-positive cell number was counted by visual recognition at the indicated anatomical brain regions.

### 4.10. Statistical Analysis

All data are presented as mean ± standard error of the mean (s.e.m.). Statistical analyses were performed using two-tailed unpaired *t*-tests and one-way analysis of variance (ANOVA), followed by Tukey’s post hoc analysis with GraphPad Prism 9 (San Diego, CA, USA). *p* < 0.05 was considered statistically significant [47].

## Data Availability

Data will be made available upon request.

## Supplementary Materials

The following supporting information can be downloaded at: https://www.mdpi.com/article/10.3390/ijms241311036/s1.
