# Supplementary material for: Torpor-like Hypothermia Induced by A1 Adenosine Receptor Agonist: A Novel Approach to Protect against Neuroinflammation
Source: Int J Mol Sci. 2023 Jul 3;24(13):11036. doi: 10.3390/ijms241311036 (PMC10341625; doi:10.3390/ijms241311036)
Supplement: Supplementary file 1 [file ijms-24-11036-s001.zip › ijms-2469040-supplementary.pdf]

Supplementary Materials

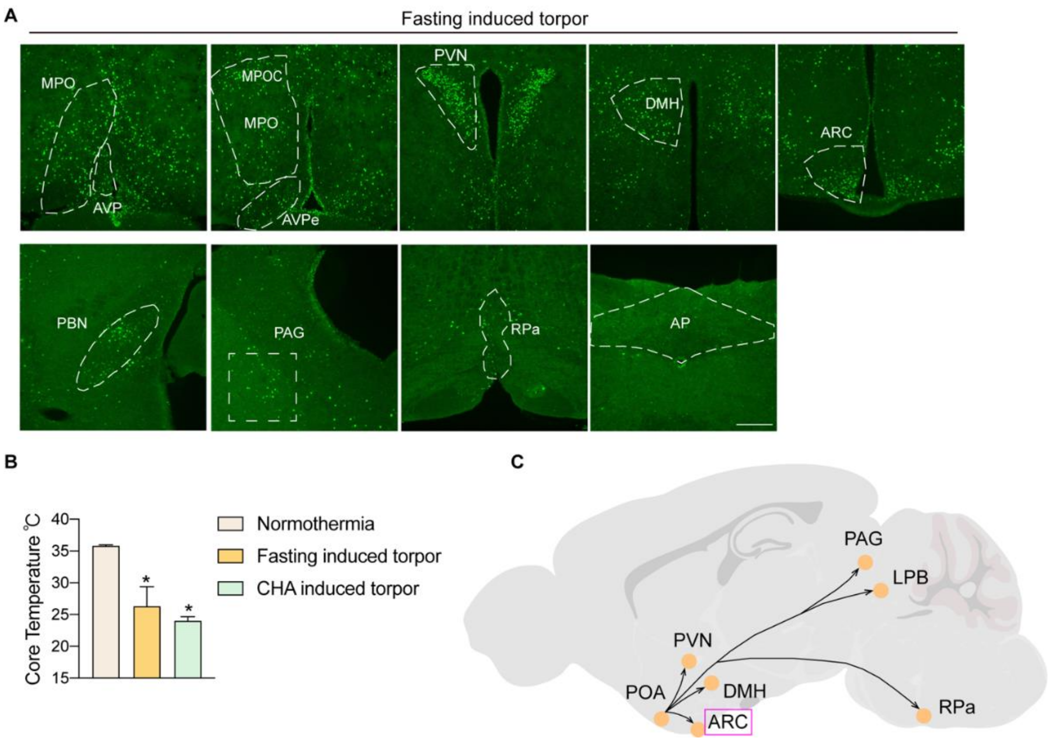

Figure S1. Whole brain tracing of neural circuit upon fasting induced torpor. (**A**) c-Fos staining of nucleus of POA, PVN, ARC, DMH, PBN, PAG, RPa, and AP. (**B**) Core temperature comparison among normothermia, fasting induced torpor and CHA induced torpor. (**C**) Illustration of neural circuit projection upon fasting induced torpor. ARC: arcuate nucleus. Data are presented as mean ± SEM. Unpaired t-test was performed in all experiments. $P < 0.05$ for *. Scale bar = 200 μm.

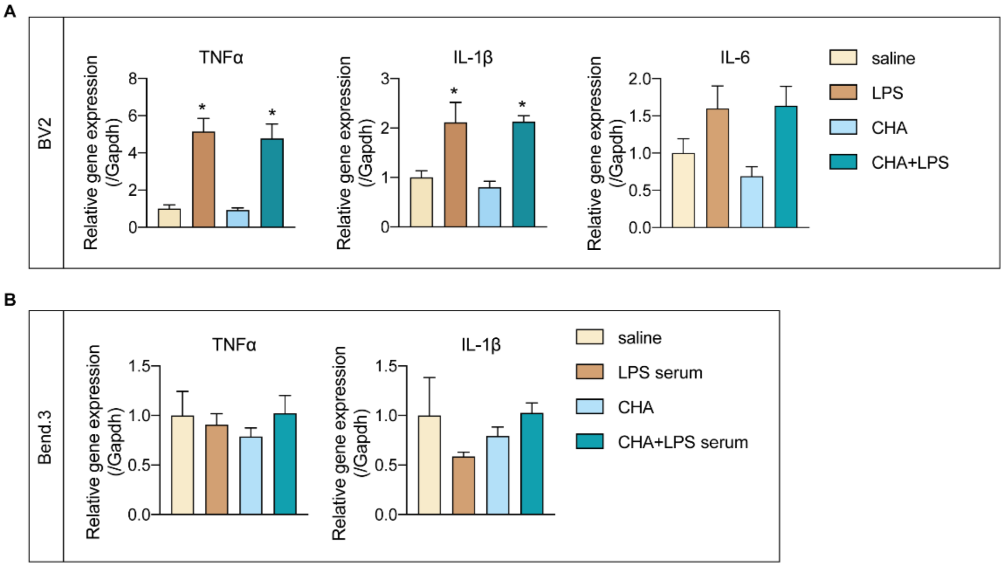

Figure S2. CHA does not have anti-inflammatory effects on microglia and brain endothelial cells. (**A**) mRNA level of proinflammatory cytokines (TNF-α, IL-1β, and IL-6) in BV2 cells at 37 ℃ after LPS stimulus with or without CHA. (**B**) mRNA level of proinflammatory cytokines (TNF-α and IL-1β) of bEnd.3 cells at 37 ℃ after LPS-serum stimulus with or without CHA. Data are presented as mean ± SEM. One-way ANOVA followed by Tukey's post hoc analysis was performed in all experiments. $P < 0.05$ for *.
